# Estimation of Dense Plasma Temperature Formed under Shock Wave Cumulation

**DOI:** 10.3390/ma13214923

**Published:** 2020-11-02

**Authors:** Valerii Sobolev, Edgar Caseres Cabana, Natalia Howaniec, Roman Dychkovskyi, Bartłomiej Jura, Andrzej Bąk, Sebastian Iwaszenko, Adam Smoliński

**Affiliations:** 1 Department of Construction, Geotechnics and Geomechanics, Dnipro University of Technology, D. Yavornytskoho Ave 19, 49005 Dnipro, Ukraine; soboliev.v.v@nmu.one; 2 Scientific Research Institute of the Center of Renewable Energy and Energy Efficiency, Universidad Nacional de San Agustin de Arequipa, San Agustin Street 107, Arequipa PE-04000, Peru; ecaceresca@unsa.edu.pe; 3 Central Mining Institute, Plac Gwarków 1, 40-166 Katowice, Poland; nhowaniec@gig.eu (N.H.); bjura@gig.eu (B.J.); siwaszenko@gig.eu (S.I.); 4 Department of Underground Mining, Dnipro University of Technology, D. Yavornytskoho Ave 19, 49005 Dnipro, Ukraine; Dychkovskyi.r.o@nmu.one; 5 Institute of Chemistry, University of Silesia, Szkolna 9, 40-007 Katowice, Poland; Andrzej.Bak@us.edu.pl

**Keywords:** plasma of high enthalpy, plasma generators, shock waves, plasma jets

## Abstract

The research was carried out by means of implosion plasma generators with conical and hemispherical compression chambers to conduct a quantitative assessment of the boundary temperature of super dense plasma jets. It was proved experimentally that nuclear transformations in metals are caused by the impact of super dense plasma jets (11, ..., 12) × 10^3^ kg/m^3^. The boundary temperature of these jets was evaluated. It was estimated that the nominal boundary temperature of the studied implosion plasma generators is 10^6^ К. The pressure in the target at the penetration of the super dense jet (~12,000 kg/m^3^) at the speed of 28,000 m / sec is more than 30 ТPa. The boundary temperature was estimated and proved to depend on the pre-determined values only slightly. It was experimentally established that stable isotopes of manganese Mn^55^ (up to 27%) are formed in iron targets as a result of high temperature plasma jet penetration. The appearance of manganese must be related to iron transformation into stable isotopes Fe^56^ and Fe^54^. The obtained results may be applied for investigating structural changes in metals under the conditions of impulsive super high temperatures and pressures. This method can be also used as a testing ground for studying the physical conditions of forming chemical elements as well as super dense plasma jets.

## 1. Introduction

The study of shock waves cumulation in gases in conical and hemispherical cavities with the use of condensed explosives as a primary energy source contributes significantly to a better understanding of extreme physical parameters of the material. An explosion of a relatively small charge (about 1 kg) results in an energy release of ~4 × 10^6^ J at the power of ~10^11^ W. Explosive plasma generators enable to obtain high-temperature plasma with high density and pressure, as evidenced by experiments with deuterium compression in conical cavities with a diameter of ~10^−3^ m. Under these conditions, the neutron yield 10^4^, ..., 10^8^ per impulse was recorded experimentally [1]. 

The authors [2,3] believe that the appearance of neutrons suggests achieving the conditions necessary not only for the gas ionization but also for the occurrence of a thermonuclear DD reaction. Helium nuclei which are absent in the initial state are synthesized in this nuclear reaction. The estimated plasma temperature in the focus of a cumulative flow in this case equals ~10^6^ K. It should be noted that under these conditions plasma is obtained only due to the gas-dynamic heating of gas by means of shock waves and due to the shock-free compression of gas [4,5,6,7]. The gas-dynamic method for high-enthalpy plasma obtained in explosive generators is a distinctive feature compared to the prevailing methods of electrical and laser heating [8,9,10].

The purpose of this research is to study the transformations in metals resulting from the influence of high-density plasma jets as well as to estimate the threshold temperature of these jets.

## 2. Materials and Methods

Conical compression chambers with the size larger than the cavity (3 mm diameter) were used in the experiments. The generator was conserved after the explosion and the structural changes and microstructural transformations of the areas burned by plasma in the “piston” and the matrix were studied. The microstructure and chemical composition of the metals used in the study were investigated using an optical microscope equipped with a micro-roentgen analyzer, whereas the content and quantity of chemical elements were studied using an electron microprobe analyzer. Explosive plasma generators with a compression chamber in the shape of circular cone and a spherical segment are investigated in this work. The working gas (air under normal conditions) was compressed and heated by a system of shock waves formed in the cavity between the moving aluminum plate and the hemispherical copper compression chamber.

**Figure 1:**
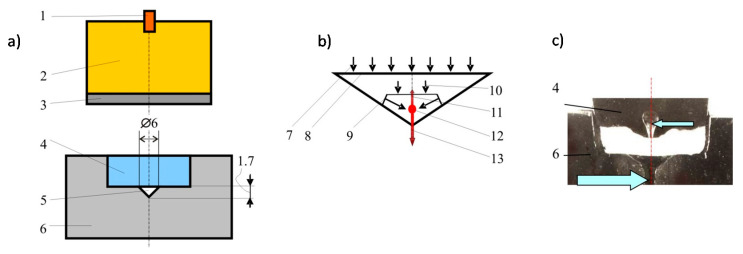
The plasma jets generator with a conical cavity: (**a**)—device diagram: 1—detonator; 2—explosive; 3—metal plate; 4—metal piston; 5—conical cavity; 6—steel matrix; (**b**)—the scheme of possible shock-wave flows in a conical hollow: 7—the direction of the particle motion; 8—the surface of the plate; 9—Mach shock wave; 10—the front of wave entering the cavity; 11, 13—plasma jets; 12—the collision of plasma streams; (**c**)—the jets burning-through the barrier; the formed cavities are indicated by the arrows; dimensions are given in mm.

The first block of experiments was conducted for explosive plasma generator with the compression chamber of the conical shape (see Figure 1). Its general overview is shown in Figure 1a.

The plasma generators with the conical compression chamber were made of steel containing С (≤0.25%), Р (0.07%), S (0.06%) and Fe. In order to conserve the generator after the blast processing, a 35 mm thick layer of lead was adjacent to the outer side surface and the lower base of the matrix. The physico-chemical transformations in the matrix material and the piston were studied after the shock-wave action.

**Figure 2:**
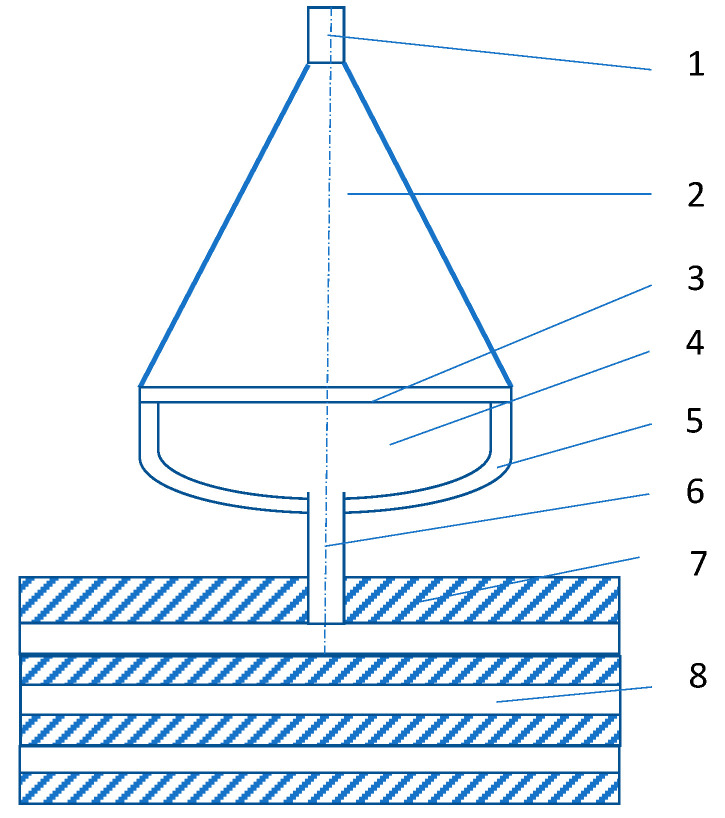
The impact of high-enthalpy plasma on the metal target: device diagram: 1—detonator; 2—explosive charge; 3—aluminum plate-piston; 4—working gas; 5—hemispherical copper compression chamber; 6—opening; 7—shield; 8—metal target.

The second block of experiments was conducted for explosive plasma generator with the compression chamber of the hemispherical camera. The interaction of the high-enthalpy plasma stream with multilayer metallic samples (1.5 mm thick alternating sheets of steel and aluminum) was studied by means of explosive plasma generators with the compression chamber in the form of a spherical segment, see Figure 2. The explosion of the detonator initiates the detonation in the explosive charge with the products of the explosion accelerating the aluminum plate-piston. The sectional view of the experimental device is demonstrated in Figure 2 (the device is axisymmetric). High-enthalpy plasma, which was formed in the focus of the flow near the top of the hemisphere, flows from the compression chamber through the opening. The shield protects the metal target from the direct impact of generator fragments.

The mass of working air in the compression chamber equals 22 mg. The total plasma energy of about 7 kJ was measured by means of the calorimetric method. The estimated plasma temperature is ~2 × 10^4^ K, whereas the plasma pressure at a typical volume of 0.25 cm^3^ is about 10 GPa with a density of the radiant heat flux from plasma of ~1 GW/m^2^.

The moving mass of high-enthalpy plasma produces mechanical and thermal shocks on the metal target. Both shock effects are very intense, as the plasma pressure exceeds the tensile strength of the target material and the thermal radiation of the plasma leads to the melt and evaporation of the target surface.

## 3. Results and Discussion

The explosion of the detonator generates the detonation wave in the charge of condensed explosive. The explosion products accelerate the metal plate. The plate is decelerated by the shock on the metal piston which is accelerated and compresses the working gas in the conical cavity located in the metal matrix. Figure 1b presents the scheme of the gas compression in the conical cavity resulting from the direct and reflected Mach shock wave going through the undisturbed gas. After the collision of the shock waves, the plasma focus is formed in the area of the cavity axis. The latest phases of the compression of the formed high-temperature plasma occur in the unstressed mode. Mach shock wave front converging with the axis of the conical cavity during the high-speed “pressing” in the cavity of the metal “piston” forms two symmetrical plasma jets coincident with the axis of the cone and directed into the piston and the matrix, that is directed in opposite directions.

Generators with a hemispherical camera safely reproduce the process of high-enthalpy plasma formation, and, for example, can be used in the experimental modeling of high-speed celestial bodies entering the Earth’s atmosphere.

The relationship between the volume of the crater formed by the impact of the plasma jet and the Brinell hardness HB of the target material was established experimentally.

The results of the plasma action on the metal parts of the conical device are shown in Figure 1c. Two channels of plasma jets pierced the metal matrix (indicated by a large arrow) and a metal stopper (indicated by a small arrow) pointing to the high pressure of plasma. One of these two channels which were formed in the matrix has a larger size. It seems natural, since in this direction the plasma under compression gets an impulse from the “piston.” The channel in the opposite direction is formed, apparently, in the dispersion of the plasma focus in the later phases of the process when the pressure noticeably decreases. It is assumed that that the formation of the symmetrical jet is determined by the collision of many streams flowing down to the top of the conical cavity surface. Along the axis in the device, the plasma flowing along the cone surface to its top will be represented by two streams whose collision generates two new jets in opposite directions. This process is similar to the jet and slug of a shaped charge.

An abnormally high value of manganese—up to 27% was revealed in the “piston” after the experiment, Figure 1, (steel which contains the impurities of Mn, Si, S in an amount up to 0.08% before the experiment) and in the matrix (steel which does not contain Mn and Si before the experiment). The spasmodic increase of Mn content regards the area outlying the “piston” from the bound zone of 32 μm, in the matrix—26 μm. The matrix consisted of stable isotopes Fe^56^ and Fe^54^, the isotopes of detected manganese—Mn^55^ are also stable. The manganese appearance involves iron transformation. The mechanism of the formation of new chemical elements under these conditions does not have a theoretical explanation. In this paper, it is assumed that their formation results from the processes involving an increase in energy density due to the redistribution of the medium energy during its motion; that is the processes occurring in collisions of dense plasma streams flowing from the surface of the conical cavity. A similar phenomenon is found in the superdeep penetration of microparticles into metals [11,12,13,14].

The pressure of 90–100 GPa in the “piston” is the result of the impact of the metal plate. Depending on the pressure in the “piston” and accordingly to the shock wave velocity entering the conical chamber, the flow of dense plasma is formed. Approaching the top of the conical chamber, the plasma flows collide resulting in the formation of high-energy plasma jets directed toward both the target and the “piston,” that is in opposite directions (Figure 1b). In plasma commutation, there is the relationship between flow rates (velocity), pressure and temperature. Previous studies have shown a fairly wide range of the results of these parameters. Unfortunately, it is not possible to take them all into account. For our study, the analysis was taken according to the known approaches, consisting in allocation of characteristic points which will establish the basic properties of work of the entire system [1,3,15]. The velocity of the jet penetration into the target is estimated at ~27,800 m/s. Using the method of calculating the parameters characterizing the ultra-high-speed cumulation, the estimate of the jet pressure is ~34.5 TPa, whereas the minimum estimated temperature is 1.3 × 10^6^ K.

**Figure 3:**
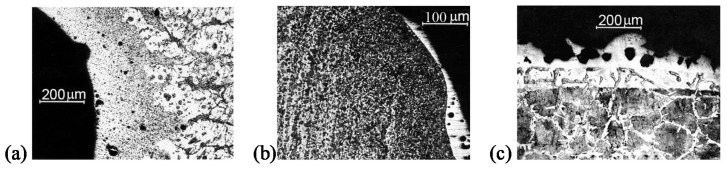
Microsection of metal surface layers after the impulse heating of the surface in contact with plasma from the explosive generator: (**a**) and (**b**)—the microstructure adjacent to the surface of the “piston” (**a**) and holder (**b**), respectively; (**c**)—the micro-section of the metal wall cut exposed to the plasma thermal shock.

The zone of structural changes with lattice micro-imperfections showing the super-high-speed cooling mode (Figure 3a) is adjacent to the surface of the mushroom-shaped cavity formed in the “piston.” At the bottom of the cavity, the width of the zone varies between 300 and 420 μm, while at the top of the cavity it is reduced to 50 μm. Obviously, the very fast heating of the matrix surface is the result of developing gas-dynamic processes. A thin white layer of non-etching metal with the width from 10 to 110 μm appeared at the edges of the cavities and along the inner surface (Figure 3b) [7]. It should be noted that the white layer is not continuous and that it has constant depth. These white layers are typically for high-speed thermal processing, such as a laser treatment. The ultrafine granularity and significant distortion of the crystal lattice are characteristic for the white layer microstructure. The dislocation density in the “piston” increased after the shock-wave compression by a factor of 10^4^ and amounted to 7.4 × 10^16^ m^−2^. The value of micro-distortions in the crystal lattice after the explosion has increased ten times: value Δa/a to the “piston” is 0.77 × 10^−3^ in the initial state and after the treatment—0.16 × 10^−2^.

The behavior of the multilayered target demonstrates the plastic motion of the material during the formation of the crater (Figure 2). The shape of the crater during the plasma impact is generally similar to that one formed under high-speed impact of a solid striker. The surface of the crater walls reveals irregular roughness (Figure 2). This is the consequence of the well-known Rayleigh Taylor instability which arises when the interface between two media of different densities is accelerating; in this case it is the plasma—metal interface [16].

The target weight before and after the experiment revealed a partial loss of the mass, corresponding to approximately 10% of the crater volume, which results from the fact that part of the target material is ejected during the crater formation. However, the mechanism of the crater formation is not the key one. In fact, the crater formation is caused by the plastic spreading of metal. The volume of the crater is inversely proportional to the Brinell hardness HB of the target material and can be represented by the formula derived on the basis of statistical data using various metals as the target:

V = C/HB(1)

where C is a constant.

Figure 3c shows the photograph of the microsection of the metal wall cut exposed to the plasma thermal shock of the explosive generator. Two surface layers with a modified crystal structure are clearly distinguished. The typical thickness of these layers is about 100 µm. The surface of the crater is covered with a light layer of the solidified melt with no visible crystal structure and a varying thickness in the range of 10–100 μm. The estimated velocity of the solidified layer cooling is more than 10^5^ K/s due to the rapid heat elimination in the metal. Amorphous metal is usually formed under such rapid cooling, which seems to be happening in these conditions. The volume of the conserved melt layer is less than 1% of the crater’s. There are many micropores in the light layer. Probably, the pores appear because of vapors release during the volume boiling of the superheated metal with the sharp decrease of the plasma pressure at the wall.

Under the layer of the solidified melt, there is a 100 µm layer of hardened metal with increased micro-hardness, formed by plasma heating of the surface and the subsequent rapid cooling by the heat sink to the deeper layers of cold metal. The initial material is under these two layers.

**Figure 4:**
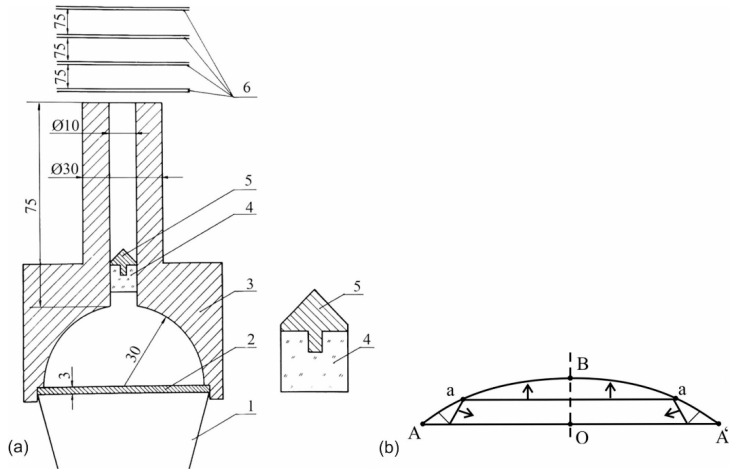
Diagram of the device for the high-enthalpy plasma throwing; (**a**)—the launched body is on the right; (**b**)—the diagram of shock fronts in the later stages of the gas compression in a spherical segment: 1—explosive charge from the alloy TNT/RDX 50/50, weight 0.6 kg; 2—copper plate; 3—steel compression chamber and the shaft, the mass of working gas is 0.14 g; 4—protective plexiglass cylinder, weight 0.8 g; 5—launched aluminum body, weight 0.6 g; 6—contact transducers; dimensions given in mm.

The explosive generator of high-enthalpy plasma with the hemispherical compression chamber was also used for high-speed throwing, see Figure 4. The height of the plexiglass cylinder is equal to the inner diameter of the shaft and its diameter provides a tight fit in the shaft that protects the launched body from the destruction by plasma. Moreover, to reduce the initial jump of the pressure, the plexiglass cylinder was set in the shaft’s bore at 5 mm from the mouth of the chamber.

The velocity of the launched body is determined by means of the two following methods: by the time of the transducers locking and with the application of an ultrafast photo-recorder. Both methods have recorded the same flight velocity of 9000 m/s.

Explosive plasma generators, Figure 4, in various modifications without a launched solid, are used to produce strong shock waves in gases and high-speed gas streams. The shock wave with a velocity of 43,000 m/s was recorded in air under normal conditions. Gas streams with even greater velocity are generated in other gases with low initial density [17].

Differences in the discussed types of explosive plasma generators are mainly determined by the structure of shock fronts and the motion of plasma streams which are defined by the geometrical shape of the compression chamber. This is the cone in the first case and the spherical segment in the other one. The structure of the main shock fronts in the cone is shown in Figure 1b. The spherical segment ABA as well as the plate-piston are presented in Figure 4b; a—represents the front of the first shock wave. A system of reflected shock and acoustic waves is formed near angle A of the spherical segment.

The flow of gas streams in the compression chamber is non-stationary, two-dimensional, with shock waves of varying intensity and ionized gas. The exact calculation of such a complex flow requires numerical modeling, which was made in References [18,19,20,21]. In these papers are proposed a simple method to estimate this flow based on the following considerations.

As long as the plate-piston AA’ covers the distance OB, the junction point A of the plate with the spherical segment covers the distances AO = OA; AB ≈ AO (Figure 4b). Point A is a geometric point (not a material one) and can be compared to the point of closing the blades of scissors. Accordingly, its velocity is the phase velocity. Assuming the proximity of the plate to the top of the segment OB < OA and using elementary trigonometric functions, we get the following estimation of the phase velocity U of point A motion to the axis (along line AO):

(2) $$U\approx \frac{1}{\sin\alpha }U_{0}\approx \frac{1}{\alpha }U_{0}$$

where *U*_0_ is the velocity of the plate, α is the angle through which the segment OA is “visible” from the center of the sphere.

The phase velocity of point A is many times higher than the velocity of the plate when the plate nears the top of the compression chamber. For example, if *U*_0_ = 4000 m/s and sin α = 0.2, then the phase velocity *U* = 20,000 m/s. Plasma, located near the corner point A, achieves a mass velocity equal to the indicated phase velocity. The specific kinetic energy of plasma is E = 0.5⋅*U*^2^ = 2 × 10^8^ J/kg, which is about 50 times more than the energy density of the explosive. The radial velocity of plasma at the plate approaching the top of the segment will grow as long as the plasma pressure begins to decelerate the plate significantly. It should be taken into consideration that the total plasma energy will be even larger due to thermal and ionization energies. The kinetic energy is converted into the thermal energy as the radial streams collide on the axis.

Thus, the arguments based on the approximate equality of the phase and mass velocities allow us to obtain some quantitative integral estimation without the knowledge about the parts of the real flow.

The estimation of the threshold temperature of explosive plasma generators can be obtained as follows. For plasma heating in explosive generators of the investigated type, the high dependence of the coefficient of radiant thermal conductivity on the temperature leads to cooling which becomes equal to heating rapidly in a narrow temperature range. As a result, further temperature growth stops. In an ideal situation, we can talk about the threshold temperature.

All further considerations are based on the data given in Reference [18].

The equation condition of the heating power and plasma cooling, that is the discontinuance of the heating mode

pu = η|grad*T*| (3)

where *T* is the temperature; η is the coefficient of thermal conductivity, p is pressure and u is the module of the velocity compression.

The radiant thermal conductivity in these conditions is the main mechanism of heat transfer for optically dense plasma in the diffusion approximation

(4) $$\eta =\frac{16}{3}\sigma \cdot T^{3}\cdot l$$

where *σ* is Stefan-Boltzmann constant and *l* is the Rosseland free path of radiation.

The Rosseland free path of radiation for gases of many-electron atoms in the area of multiple ionization is

(5) $$l=8.4\cdot 10^{-14}{\frac{T}{mp^{2}}}^{\frac{11}{2}}$$

where *m* is the average degree of ionization and *p* is pressure. To estimate, we assume |grad*T*| ≈ *T*/r (r is a characteristic dimension of plasma).

Using the indicated formulas, we estimate the threshold temperature of plasma T*

T* = 1.1 × 10^2^(*mp*^3^*ur*)^2/19^ (6)

Substituting in the last formula the typical experiment conditions with *p* = 2 × 10^10^ Pa, *u* = 4 × 10^3^ m/s, *r* = 10^−2^ m and *m* = 5, we get T* = 3.4 × 10^5^ K.

Note that this estimation of the threshold temperature depends weakly on the determinants of its value due to extracting the root of a high degree.

## 4. Conclusions

Practical studies presented in the paper are necessary for expanding the scope of plasma technologies. The authors consider the formation of the most common forms of plasma jets generator with conical and hemispherical cavities. The sample points of pressure, velocity of dense plasma and temperature formed under shock wave commutation were set. On this basis the operation of the entire system was established. The estimates of physical parameters, assuming local thermodynamic equilibrium and taking into account only the radiant cooling, suggest that the conditional threshold temperature for the investigated explosive plasma generators is about 10^6^ K.

The designed explosive plasma generators as well as the method of investigating structural changes in metals under conditions of pulsed ultra-high temperatures and pressures could be used as a unique testing ground for studying the physical conditions of high-density plasma jets formation and the nucleation of chemical elements.
